# The canine gut microbiome as a translational model for human health and aging

**DOI:** 10.1128/mbio.01951-26

**Published:** 2026-09-15

**Authors:** Zsolt Boldogkői, Dóra Tombácz

**Affiliations:** 1 Department of Medical Biology, Albert Szent-Györgyi Medical School, University of Szeged 37442, Szeged, Hungary; 2 MTA-SZTE Lendület GeMiNI Research Group, University of Szeged 37442, Szeged, Hungary; The Ohio State University, Columbus, Ohio, USA

**Keywords:** canine gut microbiome, translational model, long-read sequencing, comparative metagenomics, functional microbiomics, dysbiosis, cognitive decline, One Health

## Abstract

The domestic dog is increasingly recognized as a complementary translational model for microbiome research relevant to human health while also offering direct benefits for veterinary medicine. Dogs share environments and dietary exposures with humans and show substantial taxonomic and gene-content similarity to the human gut microbiome, although the apparent degree of similarity depends strongly on whether read recruitment, gene-catalog overlap, or functional overlap is assessed. Dogs also develop spontaneous chronic enteropathies, obesity-associated metabolic dysfunction, cancers, cognitive dysfunction, and behavioral disorders in which gut microbial changes can be examined under clinically realistic conditions. Their shorter, breed- and body-size-dependent lifespan permits repeated sampling across development, aging, disease progression, and treatment within practical timeframes. Breed structure, full-sibling cohorts, controlled dietary interventions, and access to clinically indicated samples from multiple anatomical compartments can strengthen selected study designs, although they do not provide the tighter genetic, environmental, and microbial control achievable in rodents. Here, we evaluate the research questions for which canine cohorts are especially informative, synthesize evidence linking canine and human disease-associated microbiomes, and critically assess aging, causal inference, and One Health applications. Major limitations include the need for breed and body-size stratification, heterogeneous diet and husbandry, incomplete treatment histories, the lack of routinely available gnotobiotic canine systems, and gaps in canine-specific reference resources. The canine system is therefore most informative when study designs exploit its clinical realism while acknowledging the tighter experimental control offered by reductionist models, positioning the dog as a complementary rather than a universally superior model.

## INTRODUCTION

Alterations in gut microbial community structure and function have been associated with aging and with several human diseases, including inflammatory bowel disease (IBD), metabolic disorders, cancer, and neurological disorders (1–3). These associations are often bidirectional: altered microbial functions may contribute to disease, while disease itself—as well as aging, diet, medication, and treatment—can reshape the microbiome. Distinguishing cause from consequence is therefore difficult, particularly in multifactorial chronic conditions. Integrative microbiome studies that combine metagenomics, metatranscriptomics, metabolomics, and host measurements can characterize these relationships in detail, but interventional and mechanistic studies still require suitable experimental models. No single model reproduces all aspects of human biology. Rodent models provide exceptionally tight control over genetic background, environmental conditions, and microbial colonization, the latter through gnotobiotic systems that include germ-free animals and animals colonized with a defined microbiota. By contrast, companion dogs (*Canis lupus familiaris*) develop many diseases spontaneously and receive treatment in routine clinical settings, allowing studies to capture interindividual variation, comorbidities, and longitudinal treatment responses that are difficult to reproduce in laboratory models (4, 5).

The domestic dog offers a complementary translational system rather than a universally superior alternative to other models. Comparative studies have identified taxonomic, gene-level, and diet-responsive similarities between canine and human gut microbiomes (6, 7), and dogs naturally develop diseases accompanied by microbiome alterations that partly resemble those observed in humans (8, 9). IgA responses, Peyer’s patches, and intestinal intraepithelial lymphocytes are components of a shared mammalian gut-associated immune architecture (10–12), but their presence does not constitute a canine-specific advantage over rodents. Additional strengths include well-characterized longitudinal cohorts, repeated sampling, methodological benchmarking, and access to established veterinary diagnostic procedures (13). Long-read sequencing (LRS) can improve species- and strain-level resolution in such studies (14), while genomic studies of modern and ancient wolves support the use of wolves as informative but imperfect evolutionary comparators for understanding dog domestication (15, 16).

**Fig 1:**
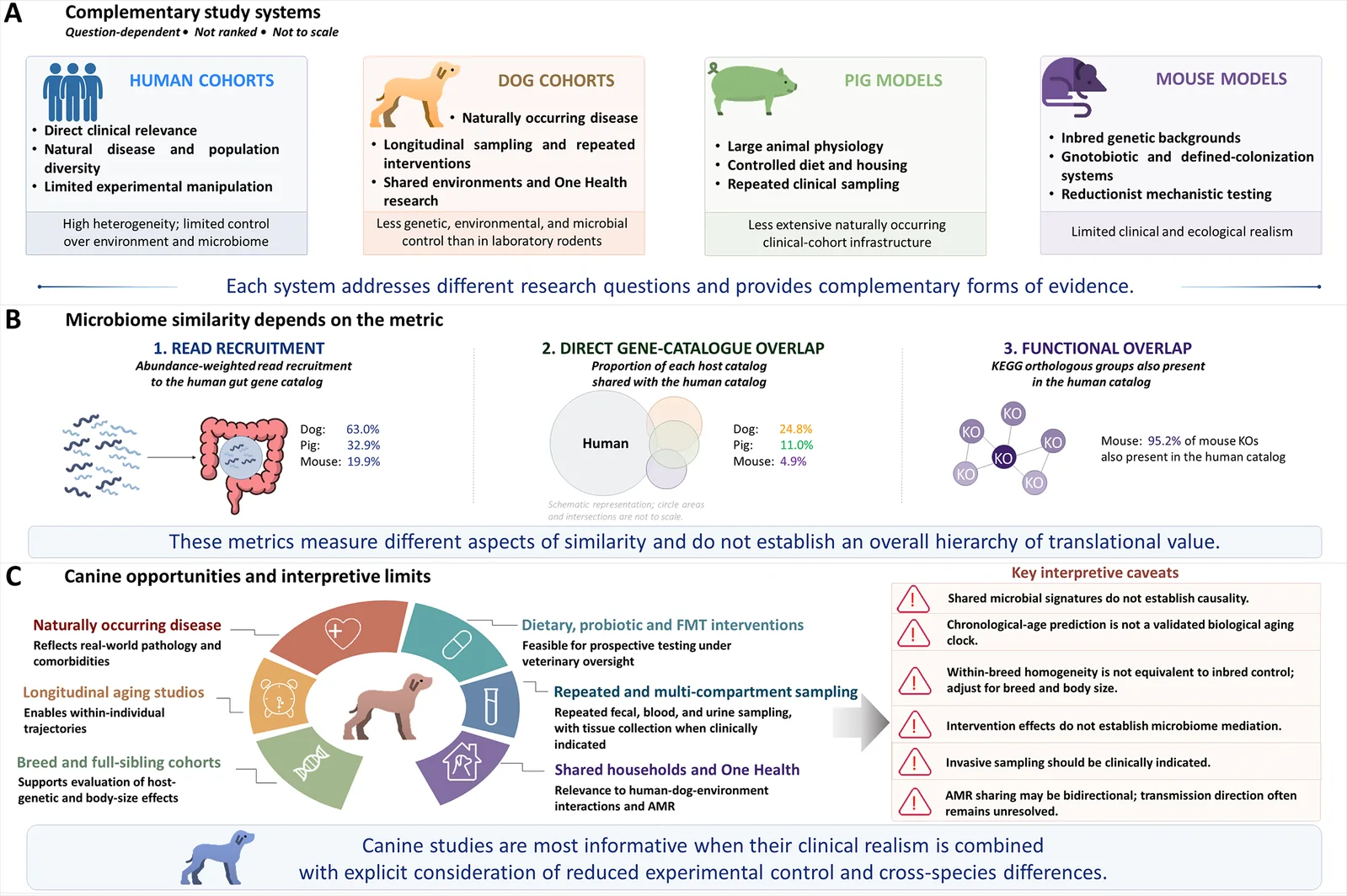
Question-dependent use of canine cohorts in translational microbiome research. (**A**) Human cohorts, dog cohorts, pig models, and mouse models provide complementary forms of evidence and are suited to different research questions. Human cohorts offer direct clinical relevance but limited experimental manipulation. Canine cohorts enable the study of naturally occurring disease, longitudinal trajectories, repeated interventions, and shared human–animal environments, but provide less genetic, environmental, and microbial control than laboratory rodents. Pig models combine large-animal physiology with controlled diet and housing, whereas mouse models provide inbred genetic backgrounds, gnotobiotic and defined-colonization systems, and reductionist mechanistic testing. The comparison is qualitative, question-dependent, not ranked, and not to scale. (**B**) Microbiome similarity depends on the metric used. Abundance-weighted read recruitment to the human gut gene catalog was 63.0% for dog, 32.9% for pig, and 19.9% for mouse samples, whereas direct gene-catalog overlap was approximately 26%, 11.0%, and 4.9%, respectively (7). Functional overlap may remain high despite lower sequence-level overlap: 95.2% of the KEGG orthologous groups identified in the mouse gut catalog were also present in the human catalog (19). These measures describe different aspects of microbiome similarity and do not establish an overall hierarchy of translational value. (**C**) Canine cohorts offer opportunities to study naturally occurring disease, longitudinal aging, breed- and full-sibling-associated variation, dietary and microbiome-directed interventions, repeated multi-compartment sampling, and One Health questions. Their interpretation requires several qualifications: shared microbial signatures do not establish causality; prediction of chronological age does not constitute a validated biological aging clock; within-breed homogeneity is not equivalent to inbred genetic control; intervention effects do not establish microbiome-mediated causality; invasive sampling should be restricted to clinically indicated procedures; and strain or antimicrobial-resistance sharing may be bidirectional, with transmission direction often remaining unresolved. AMR, antimicrobial resistance; FMT, fecal microbiota transplantation; KEGG, Kyoto Encyclopedia of Genes and Genomes; KO, KEGG orthologous group.

Population-scale shotgun metagenomic analysis from the Dog Aging Project (DAP) has also associated canine gut microbial composition with chronological age, diet, clinical measurements, and lifestyle in a diverse cross-sectional cohort of more than 900 dogs (17). Here, we examine the research questions for which canine studies are particularly informative—including naturally occurring disease, longitudinal studies of aging, intervention studies, and One Health research in shared environments—and the areas in which canine findings require confirmation in complementary rodent models and human cohorts (18). Figure 1 compares complementary study systems, distinguishes the principal metrics used to assess human–animal microbiome similarity, and summarizes the opportunities and interpretive limits of canine cohorts. Table 1 provides a structured overview of the key strengths and corresponding design considerations of the canine model.

**TABLE 1:** Key strengths and design considerations of the dog as a translational microbiome model^*a*^

Feature	Description	Relevance/example
Microbiome comparability	Dog metagenomes show high recruitment to the human gene catalog and greater sequence-level catalog overlap than mouse data sets in one comparison, while functional overlap can also be high in mice.	Supports question-specific comparison of genes and pathways; no single metric establishes overall model superiority.
Shared mucosal immune architecture	Dogs possess mammalian gut-associated immune features, including IgA responses, Peyer’s patches, and intraepithelial lymphocytes.	Supports microbiome–immune studies, but these features are not uniquely canine or absent from rodents.
Spontaneous disease models	Dogs naturally develop chronic enteropathy, obesity-associated dysfunction, cancers, cognitive dysfunction, and behavioral disorders with reported microbiome associations.	Enables clinically realistic longitudinal and treatment-response studies without experimental disease induction.
Lifespan and aging	A comparatively short, breed- and size-dependent lifespan permits repeated sampling across development, aging, frailty, and disease.	Supports lifespan-scale trajectories; chronological age associations should not be equated with a validated biological aging clock.
Breed and full-sibling designs	Within-breed cohorts and full siblings from the same litter can reduce heterogeneity relative to broadly outbred human cohorts; multi-breed studies require explicit population structure control.	Facilitates matched designs and host-genetic mapping, with a trade-off between control and generalizability.
Diet and intervention studies	Dietary changes, microbiome-directed products, FMT, and selected clinical interventions are feasible with repeated sampling.	Strengthens inference about intervention effects, but feasibility alone does not isolate microbiome-mediated causality.
Multi-compartment sampling	Repeated fecal, blood, and urine collection is feasible; mucosal biopsy and tissue sampling can be integrated when clinically indicated.	Supports region-resolved and multi-omics analyses linking microbial and host measurements.
Evolutionary comparison	Modern wolves are close living relatives that provide ecological and evolutionary comparisons, not an ancestral baseline.	Can inform domestication hypotheses when diet, geography, captivity, bottlenecks, and admixture are modeled.
Method benchmarking	Canine samples have been used to compare extraction, library preparation, short- and long-read sequencing, and bioinformatic workflows.	Quantifies technical bias and improves cross-study harmonization.
One Health relevance	Dogs and humans share households and may be exposed to overlapping microbial and AMR reservoirs.	Supports studies of potentially bidirectional strain sharing, pathobiont, resistome, and zoonotic-risk studies.

^*a*^ Biological, clinical, and methodological features supporting selected uses of the canine model, together with the principal interpretive qualification for each feature. AMR, antimicrobial resistance; FMT, fecal microbiota transplantation.

## HUMAN–CANINE TAXONOMIC AND FUNCTIONAL COMPARABILITY

Comparative metagenomic studies show that canine and human gut microbiomes share several features, but the percentages used to describe this similarity do not all measure the same thing. Coelho et al. found that 63.0% of DNA sequence reads from dog samples matched genes in the human gut gene catalog, compared with 32.9% of pig reads and 19.9% of mouse reads (7). This value describes the proportion of sequence reads that could be matched to the human catalog, not the proportion of canine microbial genes shared with humans. Because read recruitment reflects the relative abundance of the genes represented in the sequencing reads, it is conceptually different from unweighted gene-catalog overlap. When the authors directly compared the gene catalogs, they found that 309,232 of the 1,247,405 genes in the canine catalog were also present in the human catalog, corresponding to approximately 26%. The comparable proportions were 11.0% for pigs and 4.9% for mice (7).

Shared genes are only one way to compare microbiomes. Xiao et al. found that 95.2% of the functional gene groups identified in the mouse gut microbiome were also present in the human catalog (19). These groups were defined using the KEGG database, which classifies genes according to their biological functions. This finding shows that mouse and human gut microbiomes can encode many of the same functions even when their gene sequences differ. In the comparisons cited here, dogs show greater sequence-level similarity to humans, whereas the mouse catalog shows extensive overlap at the level of functional gene groups. These measurements should be interpreted separately rather than used to rank one animal model as generally better than another. Coelho et al. also suggested that some of the similarity between dog and human gut microbiomes may result from shared diet and lifestyle. This makes dogs useful for studying microbiomes in human-associated environments, but it does not mean that dogs and humans are biologically equivalent (7).

At the phylum level, *Bacillota* and *Bacteroidota* are prominent in both canine and human gut microbiomes, together with *Pseudomonadota*, *Actinomycetota*, and *Fusobacteriota* (9, 20–22). *Fusobacteriota* is often a major component of the healthy canine gut microbiome and may account for more than 10% of the community, although its abundance varies among cohorts and with diet (21, 23). In humans, however, *Fusobacterium nucleatum* is more commonly associated with colorectal cancer and IBD, and a comparable disease association has not been established in dogs (24–27). This difference illustrates that the abundance of a taxon may have distinct ecological and clinical meanings across different host species. Similarity at a high taxonomic level, therefore, does not necessarily imply a shared role in health or disease.

Frequently detected genera in the canine gut include *Fusobacterium*, *Bacteroides*, *Prevotella, Blautia*, *Megamonas*, *Streptococcus*, *Faecalibacterium*, and *Peptacetobacter*, although their prevalence and relative abundance vary with age, diet, breed, geography, and the methods used to profile the microbiome (28–30). In a longitudinal cohort of Hungarian Pumi dogs, full-length 16S rRNA sequencing revealed age-related microbiome maturation and identified *Peptacetobacter hiranonis* (formerly *Clostridium hiranonis*) as a prominent adult taxon (14). Because these observations were obtained from a single breed, they should not be regarded as universal features of the canine gut microbiome. Independent longitudinal studies in other breeds and populations are needed to assess their broader relevance, alongside the larger, multi-breed but cross-sectional microbiome analysis published by the DAP (17).

## DISEASE CONTEXTS AND MICROBIOME ASSOCIATIONS

Dogs naturally develop a range of clinically relevant conditions—including chronic enteropathies, obesity-associated metabolic dysfunction, cancers, cognitive decline, and behavioral disorders—allowing microbiome changes to be followed during disease progression and treatment in routine veterinary practice (8, 9, 31–43). Not every canine condition is equally informative for the corresponding human disease. A meaningful comparison requires similarities not only in the diagnostic label but also in clinical features, microbiome changes, host responses, and disease course. Because the available evidence varies among conditions and is mostly observational, microbial patterns shared by dogs and humans should be viewed as candidate biomarkers or clues to possible mechanisms rather than as proof of causation.

Canine chronic inflammatory enteropathy (CIE), a condition with clinical and biological similarities to human IBD, is the best-studied example. Dogs with CIE have been reported to show reduced microbial diversity or stability, lower abundance of taxa such as *Faecalibacterium* and *Blautia*, expansion of *Enterobacteriaceae* and other *Pseudomonadota*, altered bile-acid metabolism, and widespread changes in the fecal metabolome (8, 9, 44–47). Similar alterations have been described in human IBD, although network analyses also reveal host-specific patterns of dysbiosis rather than a single disease-associated microbiome shared across species (45). A quantitative PCR-based canine dysbiosis index correlates with clinical severity and may change during treatment. Longitudinal studies have also shown parallel changes in microbial composition, unconjugated bile acids, and disease activity (48, 49). These findings support the use of canine chronic enteropathy for biomarker and treatment-response studies, but they do not establish whether dysbiosis contributes to disease, results from it, or reflects medication and dietary changes.

Canine obesity provides a different metabolic comparison. Obesity and obesity-associated metabolic dysfunction are common in dogs and share several features with human metabolic disease, including insulin resistance, dyslipidemia, and low-grade inflammation, although dogs do not consistently develop the full spectrum of human metabolic syndrome (33, 50, 51). Weight-loss interventions and controlled changes in dietary macronutrient composition can alter the canine gut microbiome and fermentation-related metabolites (7, 32, 52–54). Some microbial functions also respond to dietary changes in similar ways in dogs and humans, supporting the use of canine studies to examine how the gut microbiome responds to diet. Interpretation remains difficult, however, because diet, breed, body condition, and the degree of caloric restriction may all influence the observed changes. Moreover, no single, reproducible obesity-associated microbiome signature has been identified consistently in either dogs or humans.

Comparative oncology allows spontaneous tumors to be studied in dogs with intact immune systems, naturally occurring comorbidities, and clinically realistic treatment courses (34, 35, 42, 43, 55). In a recent pilot study of dogs receiving anti-EGFR/HER2 immunization, pretreatment gut microbial features were associated with survival (56). This finding suggests that canine immuno-oncology trials may help identify microbiome biomarkers of clinical outcome. However, the small cohort and observational design do not establish that the microbiome influenced the response to treatment. Osteosarcoma is particularly relevant because it is far more common in dogs than in humans and shares important clinical and molecular features across species (34, 35, 43). The availability of spontaneous canine cases facilitates longitudinal sampling and the early evaluation of new treatments. Direct evidence linking the gut microbiome to canine osteosarcoma remains limited, but these patients provide a valuable clinical setting in which such hypotheses can be tested.

The gut–brain axis represents another area of interest. Canine cognitive dysfunction syndrome shares behavioral and neuropathological features with age-related neurodegenerative disorders in humans (36, 37), and gut microbiome composition has been associated with age and memory performance in companion dogs (57). Earlier studies linked microbial community structure to aggression toward other dogs, phobic and aggressive disorders, and behavioral traits in working dogs (39–41). Pellowe et al. extended this work by reporting associations between several bacterial taxa, including *Blautia*, and anxiety and aggression scores (38). However, the behavioral measures, study populations, and microbial findings differ among studies, and the reported associations may also be influenced by medication, diet, age, and reliance on owner-reported behavior. The available evidence supports further longitudinal and interventional studies, but it does not yet identify a specific microbial cause of canine or human neurobehavioral disease.

## MICROBIOME DYNAMICS ACROSS THE CANINE LIFESPAN

The canine gut microbiome changes across the lifespan, with rapid maturation during early development and less consistent patterns of change in older age. Because dogs have a shorter lifespan than humans, with marked differences among breeds and body-size groups, they can be sampled repeatedly from birth to old age within a practical study period. Early microbial colonization is shaped by maternal exposure, environment, diet, and mode of birth. In a longitudinal cohort of Hungarian Pumi dogs, early microbial composition differed according to birth route, although the cesarean-delivered puppies represented only a small subgroup. Weaning was accompanied by increasing diversity and the development of a more anaerobe-rich, adult-like community.

*Peptacetobacter hiranonis* became prominent in adult animals (14). A related analysis of early-life microbiome development is currently available as a bioRxiv preprint (58). The dense sampling provides detailed information on changes within individual dogs, but these observations come from a single breed. The reported effects of delivery mode, the timing of microbiome maturation, and the prominence of particular taxa therefore require confirmation in other breeds and populations. The DAP provides a broader, multi-breed comparison, although its published microbiome analysis was cross-sectional and used one first-year sample from each dog rather than repeated samples collected across the lifespan (17).

Current evidence does not support a single, consistent pattern of microbiome change in older dogs. Fernández-Pinteño et al. found no significant differences in Chao1 or Shannon alpha diversity among age groups and reported a higher relative abundance of *Faecalibacterium* in senior dogs. The relative proportion of fecal butyrate was lower in older animals, but its absolute concentration did not differ significantly (59). Other studies have reported different findings. Kubinyi et al., for example, found that *Fusobacteriota* was less abundant in older dogs and that memory performance was associated with the relative abundance of *Actinomycetota* (57). In the DAP cohort, microbial composition and community uniqueness were associated with chronological age and supported a population-level model of age prediction (17). Because the analysis was cross-sectional, however, the model predicts chronological age but does not establish a biological aging clock or show that differences between predicted and actual age reflect healthspan. A microbiome-associated inflammaging process remains a plausible hypothesis, but it has not yet been demonstrated in dogs. Testing this hypothesis will require longitudinal studies combining microbiome profiles with inflammatory markers, intestinal-barrier measurements, microbial metabolites, frailty, cognition, and clinical outcomes.

Dogs are particularly useful for aging research when studies take advantage of their shorter lifespan and naturally occurring burden of age-related disease. They may develop several chronic conditions, receive veterinary treatment, and experience varied environmental exposures over a timescale that permits prospective follow-up. This makes it possible to examine microbiome stability, responses to illness or antibiotics, and recovery after dietary or pharmacological interventions. Differences in lifespan among breeds and body-size groups may also be informative, provided that these variables are explicitly included in the study design and analysis. With repeated microbiome sampling, longitudinal programs such as the DAP are well positioned to relate microbial changes to healthspan, frailty, disease onset, and survival (60). Canine rapamycin trials further show that interventions targeting aging can be evaluated prospectively in companion dogs (61). The main value of the canine model is therefore not simply that dogs age over a shorter period than humans, but that longitudinal microbiome studies can examine how microbial changes accompany declining health and the development of age-related disease.

## BREED STRUCTURE: OPPORTUNITIES AND DESIGN CONSTRAINTS

The genetic structure of domestic dog breeds can support both host-genetic mapping and more controlled comparisons, but it also creates important limitations. Within a single breed, genetic variation is lower than in broadly outbred human populations. Full siblings from the same litter may also share parts of their early-life environment, making them useful matched controls. They should not, however, be treated as equivalents of monozygotic twins. Carefully designed within-breed studies may improve the detection of effects associated with diet, lifestyle, medication, or disease, but they do not provide the near-uniform genetic background of inbred mouse strains or the controlled microbial colonization provided by gnotobiotic systems.

Breed-associated genetic variation and disease predispositions can provide opportunities to examine relationships among host genetics, microbiome composition, and disease susceptibility. In the Norwegian Lundehund, host genetic background was associated with gut microbiome composition, and the observed dysbiosis may be related to the breed’s high incidence of protein-losing enteropathy (62). This example illustrates how breed-associated diseases can generate hypotheses about interactions among host genetic variation, microbial community structure, and disease, although the findings require validation in other breeds and populations. The same breed structure can, however, confound multi-breed studies because breed often varies together with body size, diet, geography, and disease prevalence. Multi-breed analyses should therefore account explicitly for breed, for example, through stratification, blocking, or its inclusion as a fixed or random effect. Adequate sample sizes within each breed and replication in independent cohorts are also important. Thus, the greater homogeneity of within-breed cohorts comes at the cost of reduced generalizability, whereas multi-breed studies require larger and more carefully balanced designs.

## PRACTICAL ADVANTAGES AND TRACTABILITY

Dogs offer several practical advantages for microbiome research. Breed-related differences in lifespan and disease susceptibility, together with detailed veterinary records and expanding biobank resources, support both retrospective and prospective studies with extensive clinical phenotyping. Diet can be standardized or deliberately modified, and large metagenomic studies have linked canine gut microbiome composition to dietary protein and carbohydrate content, food format, and other nutritional factors (54, 63, 64). The body size of dogs permits repeated collection of fecal, blood, and urine samples, supporting longitudinal and multi-compartment analyses. When clinically justified, mucosal biopsies or tumor tissue collected during diagnostic or therapeutic procedures can also be included. Endoscopic sampling can reveal regional differences within the gastrointestinal tract and enables microbial profiles to be integrated with host transcriptomic, proteomic, metabolomic, and immune measurements. These opportunities do not reduce the ethical obligations of research involving companion animals. Studies require appropriate veterinary ethical review and informed owner consent, and animal burden must be minimized. Invasive research sampling should therefore be restricted to procedures that are already clinically indicated for the individual animal.

## MODERN WOLVES AS AN EVOLUTIONARY COMPARATOR

Modern gray wolves (*Canis lupus*) are the closest living wild relatives of domestic dogs, but present-day wolf populations are not their direct ancestors. Ancient-genome analyses show that dogs are more closely related to ancient wolves from eastern Eurasia than to those from western Eurasia, while also identifying western Eurasian wolf-related ancestry in some dog populations (16). These findings point to a complex origin involving more than one ancient wolf population, but the precise geographic and population origins of domestic dogs remain unresolved.

Nevertheless, comparisons between wolves and dogs can help identify microbiome features associated with domestication, adaptation to starch-rich diets, dietary change, and increased exposure to human-associated environments (65). Such comparisons should not be interpreted as a simple contrast between pre- and post-domestication states. Modern wolf populations have been shaped by demographic bottlenecks and other population changes, geography, diet, access to anthropogenic food sources, and ongoing gene flow between wolves and dogs (15). Captivity provides an additional source of variation: one small metagenomic study found that the taxonomic and functional composition of the gut microbiome of captive wolves was more similar to that of domestic dogs than to that of wild wolves (66). Comparisons involving wild and captive wolves, village dogs, and modern breeds are therefore most informative when differences in ecology, diet, geography, antibiotic exposure, captivity, and host genomic ancestry are explicitly considered.

## REAL-WORLD COMPLEXITY AND LIFESTYLE HETEROGENEITY

Dogs combine greater clinical and environmental realism than tightly controlled laboratory models with more scope for prospective standardization and intervention than most human cohorts, although their value depends on the research question. Their gut microbiomes are shaped by varied diets, medications, exposure to pathogens, aging-related changes, and complex household and social environments. Many of these exposures can be recorded prospectively, while selected variables—particularly diet and sampling schedules—can be standardized or experimentally controlled more readily than in most human cohorts. The diversity of canine populations, ranging from household companion dogs to working and rural dogs, can be informative when metadata are detailed, hypotheses are defined in advance, and major covariates are included in the analysis. This heterogeneity, however, reduces experimental control and may obscure smaller effects. Dogs are therefore well suited to studies of naturally occurring disease, longitudinal sampling, repeated interventions, and host–environment interactions, but they are less suitable than inbred or gnotobiotic rodents for isolating individual microbial mechanisms.

## FROM ASSOCIATION TO CAUSALITY

Several interventions can be studied prospectively in dogs, allowing stronger inference than cross-sectional comparisons alone. Controlled and reversible switches between commercial diets, changes in macronutrient composition, or alterations in feeding schedules permit within-dog and crossover analyses of diet–microbiome responses (7, 32, 54). Probiotics, prebiotics, synbiotics, and postbiotics can likewise be assessed using repeated clinical and molecular measurements (53). Fecal microbiota transplantation (FMT) has also been investigated in canine gastrointestinal disease (67). Recent clinical guidelines for companion animals address donor screening, administration, indications, and safety, while noting that the available veterinary evidence remains limited and varies by condition (68). In canine oncology, prospective trials can examine whether pretreatment microbial profiles predict outcome and whether microbiome-directed adjuncts influence treatment tolerance or response (56, 69).

The feasibility of these approaches does not, however, establish that their clinical effects are mediated by the microbiome, nor does it isolate a single microbial mechanism. Dietary changes affect both host physiology and the gut microbiome, probiotics may act directly on the host, and FMT transfers a complex, and only partly characterized donor community rather than a defined microbial consortium. Observed clinical effects therefore cannot automatically be attributed to a specific microorganism or microbiome-mediated pathway. In addition, dogs do not provide the routinely available germ-free and gnotobiotic systems used for controlled colonization experiments in mice. Observed microbiome–outcome associations may reflect reverse causation. Apparent treatment effects may also arise from natural fluctuations in disease severity, regression to the mean, concomitant medication, or clinical improvement without sustained engraftment of donor strains. Stronger causal inference in canine studies will require randomization where feasible, crossover or matched longitudinal designs, sham or standard-care controls, appropriate washout periods, strain-resolved donor–recipient tracking, multi-omics profiling, and mediation analysis. Such designs can strengthen causal inference about whether an intervention affects clinical outcomes, but more controlled mechanistic systems—including gnotobiotic rodents, defined microbial consortia, and *in vitro* host–microbe models—may still be needed to identify the microbial taxa, metabolites, or molecular pathways that mediate the effect.

## THE DOG AS A METHODOLOGICAL TESTBED

Microbiome profiles are strongly influenced by DNA extraction, library preparation, sequencing technology, and bioinformatic analysis, all of which can introduce systematic bias and complicate comparisons among studies (13). Canine fecal samples have therefore been used to benchmark laboratory and analytical workflows. Comparisons of extraction methods, library protocols, sequencing platforms, and taxonomic pipelines have shown that these choices can substantially affect estimated community composition and reproducibility (13, 70). The use of matched canine fecal material across extraction, library preparation, and sequencing workflows provides a basis for estimating technical variation independently of between-sample biological variation (70).

Long-read sequencing (LRS), including full-length 16S rRNA sequencing and long-read metagenomics, can improve species-level and, in some settings, strain-level resolution compared with short-read amplicon sequencing (71). In a longitudinal cohort of Hungarian Pumi dogs, full-length 16S rRNA sequencing identified *Peptacetobacter hiranonis* as a prominent adult taxon, whereas it was not detected in matched short-read V3–V4 data sets (14). This result illustrates how the sequenced region, read length, and analytical workflow can influence taxon detection, but it does not show that the species is universally abundant in dogs. High-quality canine genome assemblies further support integrated analyses of host whole-genome and microbial metagenomic data (72).

Metatranscriptomics and metabolomics provide functional information beyond taxonomic composition by characterizing microbial gene expression and metabolic output. Fecal samples do not fully represent mucosal or small-intestinal communities, but endoscopic samples obtained during clinically indicated procedures can provide information from different regions of the gastrointestinal tract. When combined with host transcriptomic, proteomic, metabolomic, and immune measurements, such samples enable comparisons across gastrointestinal regions and integrated multi-omics analyses that are difficult to perform in human cohorts (73).

## CROSS-CUTTING LIMITATIONS OF THE CANINE MODEL

Canine microbiome studies face several cross-cutting limitations. Breed, body size, relatedness, and real-world exposures introduce substantial biological and environmental variation. Findings from a single breed, kennel, or geographic population may not generalize to other dogs or to humans, whereas multi-breed studies are complicated by population structure and usually require larger, carefully balanced cohorts. Even in carefully matched canine cohorts, researchers have less control over genetic background and environmental exposures than in inbred laboratory rodents, while controlled manipulation of microbial colonization is far less feasible than in gnotobiotic rodent systems.

Translation across host species also requires caution. Taxonomic similarity does not necessarily imply functional equivalence, and sequence-level gene overlap, abundance-weighted read recruitment, and pathway-level functional overlap measure different aspects of microbiome similarity (7, 19). Fecal samples provide only a partial view of the gastrointestinal microbiome and may not accurately represent regional or mucosa-associated communities (74). Species-specific differences in intestinal physiology and microbial ecology may further alter the meaning of otherwise similar taxonomic patterns. Moreover, diet composition and food processing vary widely among companion dogs (63, 64) and often differ from typical human diets, limiting direct extrapolation of diet–microbiome relationships and findings related to obesity or microbial energy metabolism (75). Coprophagy may introduce transient or exogenous microorganisms and should be recorded where possible (76). Treatment histories, including exposure to antibiotics, antiparasitic drugs, probiotics, and other supplements, may also be incomplete or dependent on owner recall. Although canine-specific reference catalogs are expanding, incomplete representation of low-abundance and previously uncharacterized taxa can still limit taxonomic and functional annotation. Detailed metadata, appropriate adjustment for major covariates, replication across breeds and populations, and cautious extrapolation to humans are therefore essential.

## ONE HEALTH IMPLICATIONS

The close relationships among dogs, humans, and their shared environments make companion dogs particularly relevant to the One Health framework, which recognizes the interdependence of human, animal, and ecosystem health (18, 77). Household studies have shown that dogs and their owners may carry the same or closely related bacterial strains (78–80). This is relevant not only to recognized zoonotic pathogens but also to pathobionts—members of the normal microbiota that can cause disease under particular conditions. Certain *Escherichia coli* lineages, for example, can colonize the intestine without causing disease but cause urinary or other extraintestinal infections after reaching a susceptible anatomical site. Whole-genome studies have shown that some uropathogenic *E. coli* lineages occur in both dogs and humans and have identified closely related resistant *Enterobacterales* in companion animals and people living in the same household (81, 82). Such findings do not show that colonization caused disease, and genomic relatedness alone cannot establish direct transmission or its direction. They do, however, support the household as an important unit for epidemiological investigation.

Antimicrobial resistance can circulate in multiple directions. Antimicrobial use in veterinary or human medicine can select for resistant organisms, while food, direct contact, wastewater, household surfaces, and other environmental reservoirs may contribute to their spread (79–83). Resolving the direction of transmission generally requires repeated sampling of dogs, people, and potential environmental sources. Joint surveillance of the canine microbiome and resistome—the full complement of antimicrobial resistance genes in a microbial community—is therefore most informative when dogs, owners, and the household environment are sampled together and antimicrobial exposure is documented in detail. Raw meat-based diets have been associated with higher fecal carriage of *Salmonella*, *Campylobacter*, extended-spectrum β-lactamase (ESBL)-producing *E. coli*, and other antimicrobial-resistant *E. coli*, although the organisms detected and the strength of these associations vary among studies (84, 85). This may be of particular concern in households with children, older adults, or immunocompromised individuals. For selected pathogens and resistance determinants, dogs may also serve as sentinels of exposures shared across household and outdoor environments.

## OUTLOOK

Future canine microbiome studies should align their claims with the strengths and limitations of the study design. Large cross-sectional cohorts are useful for identifying reproducible associations and characterizing population heterogeneity, whereas breed-restricted longitudinal studies can follow changes within individual dogs over time. Randomized and crossover trials are better suited to testing clinical interventions, while rodent and *in vitro* systems remain important for isolating specific molecular mechanisms. In aging research, microbiome-based prediction of chronological age should be complemented by repeated measurements of inflammation, intestinal-barrier function, metabolites, age-related vulnerability, cognition, and survival. Disease studies would benefit from sampling before treatment and, in prospective cohorts, before clinical diagnosis, together with clearly defined outcomes and replication across breeds and clinical centers.

Consistent metadata collection will be equally important. Breed, body size, age, sex, and reproductive status, diet composition, and food processing, housing, activity, coprophagy, and exposure to antibiotics, antiparasitic drugs, probiotics, and other medications should be recorded in a standardized manner. Expanded canine gut microbial genome catalogs, long-read and strain-resolved metagenomics, metatranscriptomics, metabolomics, host genomics, and immune phenotyping can help connect microbial composition and gene expression with metabolic activity and host physiology. The Dog Aging Project and other prospective cohorts provide an important foundation, but their value will depend on repeated longitudinal sampling and harmonized protocols that allow population-level associations to be evaluated for predictive and mechanistic relevance (17, 60). Future work should also examine microbial communities beyond fecal samples, including those associated with the intestinal mucosa, oral cavity, skin, respiratory tract, and urinary tract, particularly in studies of clinical disease and One Health transmission.

Dogs should not be regarded as substitutes for human cohorts or as uniformly superior to laboratory models. Their main value lies in the combination of shared environments with humans, naturally occurring disease, a shorter and highly variable lifespan, breed structure, detailed veterinary phenotyping, and the feasibility of longitudinal sampling and controlled interventions. When these features are used with appropriate attention to confounding, limited experimental control, and cross-species differences, canine studies can help connect mechanistic microbiology with real-world disease and aging while also advancing veterinary care.
